# Associations Between Klotho Levels, *KL-VS* Heterozygosity and Cognition in Schizophrenia

**DOI:** 10.1093/schizbullopen/sgae030

**Published:** 2024-12-25

**Authors:** Vijaya Majumdar, Prosenjeet Chakroborty, Rashmi Arasappa, K Murugesh, Shanthala Hegde, Amrutha Jose, N K Manjunath, Arpitha Dharmappa

**Affiliations:** Molecular Bioscience and Gerosceince lab, Swami Vivekananda Yoga Anusandhana Samsthana, Bangalore, Karnataka 560105, India; Molecular Bioscience and Gerosceince lab, Swami Vivekananda Yoga Anusandhana Samsthana, Bangalore, Karnataka 560105, India; Department of Psychiatry, National Institute of Mental Health and NeuroSciences, Bangalore, Karnataka 560029, India; Molecular Bioscience and Gerosceince lab, Swami Vivekananda Yoga Anusandhana Samsthana, Bangalore, Karnataka 560105, India; Department of Clinical Psychology, National Institute of Mental Health and NeuroSciences, Bangalore, Karnataka 560029, India; Biostatistics, ICMR-National Institute of Immunohematology, Mumbai, Maharashtra 400012, India; Molecular Bioscience and Gerosceince lab, Swami Vivekananda Yoga Anusandhana Samsthana, Bangalore, Karnataka 560105, India; Molecular Bioscience and Gerosceince lab, Swami Vivekananda Yoga Anusandhana Samsthana, Bangalore, Karnataka 560105, India

**Keywords:** Klotho, KL-VS, schizophrenia, cognitive subtypes

## Abstract

**Background and Hypothesis:**

The relationship between Klotho and cognitive dysfunction in schizophrenia has been scarcely explored, with a few paradoxical findings. Hence, we aimed to enhance our understanding by testing associations between the functional KL-VS gene variant and circulating protein levels.

**Research Design:**

This case-control study included 239 healthy controls and 241 patients with schizophrenia, who were comprehensively characterized by neurocognitive tests and further subtyped into cognitive clusters; cognitively deficient (CD) and cognitively spared (CS), using *K*-means cluster analysis. Linear regression models were run to assess the main and iinteraction effects of the KL-VS heterozygosity (KL-VS^Het+^)/KL levels with confounding variables (disease status and age) on cognitive scores.

**Results:**

There was no main effect of KL-VS^Het+^ on the cognitive domains, but the CD cluster exhibited strong negative interactions between disease status and Klotho for executive function at the gene level, KL-VS^Het+^ × disease status, β = −.61, *P* = .043, with comparatively higher effect observed for KL levels, KL levels × disease status, β = −.91, *P* = .028. There was an opposing positive interaction for response inhibition, KL-VS^Het+^ × disease status, limited again to the CD cluster, β = .35, *P* = .046, with a higher effect at protein levels, KL levels × disease status, β = .72, <.004, though without CD cluster effect.

**Conclusions:**

Overall, these dissociable patterns of association across cognitive domains indicate the need to exert caution over accepting any generalised direction of effect of Klotho at gene or protein level on cognition in schizophrenia.

## Introduction

Cognitive deficits constitute the core facets of schizophrenia, with reported estimates of manifestation amounting to ~98% among diagnosed patients,^1^ spread across varied domains, encompassing attention, processing speed, memory, language, and executive function.^2,3^ However, given the consistent failures of known pro-cognitive drugs targeting different neurochemical systems, schizophrenia-associated cognitive impairments remain an important biomedical challenge from the treatment purview.^4,5^ The heritability of these deficits and their stability across the disease course allows them to be used as traits and intermediate endophenotypes in genetic studies to unravel underlying key genetic variants^6–10^ that could further guide a precision-medicine approach to the therapeutics.^11^ Moreover, earlier onset of these deficits even before the clinical onset of the disease could pave the foundation for early targeted interventions for better disease prognosis.^3^

Mechanistically, the schizophrenia-risk genes linked to cognitive dysfunction have been reported to be enriched for pathways including long-term potentiation/*N*-methyl-d-aspartate (NMDA) receptor hypofunction,^12,13^ glutaminergic and dopaminergic neurotransmitter systems,^14^ and schizophrenia-related structural and functional brain changes.^15^ There is also a reported relevance of myelination and inflammation-driven pathways as drug-target avenues for neurocognitive and psychosis severity, apart from the neurotransmitter disturbances.^4^ All of these considerations align well with the genetic candidature of Klotho (KL), an anti-aging and cognition-enhancing gene for cognitive impairment in schizophrenia. Experimentally, the elevation of KL has been associated with learning and memory domains of cognition underlined by enhanced NMDAR-related functions, and FBJ murine osteosarcoma viral oncogene homolog (FOS) expression following a learning and memory task^16^ coupled with postsynaptic enrichment of the NMDAR subunit GluN2B in the hippocampus and frontal cortex. Further, KL has also been shown to be necessary for oligodendrocyte maturation and myelin integrity.^17^ At the population level, the cognition-enhancing effects of KL have been primarily established with the heterozygosity of its life-extending genetic variant, termed KL-VS, also recognized for its influence on enhanced levels of circulating KL levels.^16^ KL-VS is a common haplotype that consists of 6 missense variants in the human *Kl* gene,^18^ represented primarily by the 2 single nucleotide polymorphisms with amino acid substitutions: V and S alleles of these SNPs, respectively, F352V (rs9536314) and C370S (rs9527025).^18^ Heterozygosity of the KL-VS variant has also been associated with greater right frontal lobe volumes,^19^ the region known to be atrophied in schizophrenia.^20^ On a contrary note, the growing literature has added ambiguity and inconsistency to the effects reported for KL-VS heterozygosity for cognitive measures at the population level,^16,21,22^ along with reported disadvantages of KL-VS heterozygote on white matter volume.^23^

The initial findings by Dubal et al. implicated the heterozygous advantage exhibited by the variant over 3 independent cognitively elderly samples (*n* = 718),^24^ independent of age. On the contrary, the emerging trends of genetic association between the KL-VS variant and cognition present mixed insights, with the modulatory influence of age, gender, and education and under specific disease pathophysiology.^10,21,25^

We find scarce evidence for studies on the association between Kl levels, the KL-VS variant, and cognitive dysfunction in schizophrenia.^10,26–30^ In 2018, Morar et al. reported a paradoxically reversed directionality of KL-VS heterozygosity with heterozygotes performing worse than noncarriers,^10^ concerning memory impairment in schizophrenia in a Western Australian Cohort and attributed the same to the pathogenic milieu of the disease.^10^ These inconsistencies are further compounded by the disparity reported in the allele frequency distribution of the KL-VS variants across varied ethnicities, in particular among different Asian ethnicities,^31–33^ hindering the straightforward generalization of genetic association-based implications.^10,18,33^ Given the above considerations, we undertook an examination of Klotho both at the protein and genetic level to delineate the association of KL-VS heterozygotes with cognitive outcomes in schizophrenia in Asian Indians, while also attempting the validation of the findings by Morar et al.^10^

## Materials and Methods

### Participants

The present study sample comprised 239 SZ patients (48.53% male, age range 18-60 years) and 243 healthy controls (HCs; 45.27% male, age range 18-60 years) of Asian Indian descent. The institutional ethics committees approved the study. Study participants signed informed consent forms before enrollment per the Declaration of Helsinki.

The patients were recruited from the National Institute of Mental Health and Neurosciences outpatient department, Bengaluru, India. Their diagnosis of schizophrenia was ascertained with the Structured Clinical Interview for DSM-IV-TR Axis I Disorder, patient edition.^34^ The controls were recruited by random sampling from local communities and screened for psychopathology to exclude those with a personal or family history of psychotic illness. All participants were assessed for cognitive performance using a neurocognitive battery spanning the vital cognitive domains (processing speed, attention, executive function, episodic memory, and response inhibition) with reported evidence of heritability.^35,36^ We used the digit symbol substitution test (DSST) to assess processing speed.^37^ The executive function domain included sub-domains working memory (WM) and verbal fluency (VF). Working domain was assessed through the letter-number sequencing task from the Wechsler Memory Scale^38^ and the verbal N-back test.^39^ Verbal fluency was assessed with the Controlled Oral Word Association Task for verbal fluency. For the verbal N-back tests, accuracy was calculated for all 2 block periods of the N-back task and then averaged to derive a mean accuracy score for each participant. Attention was scored using the color trial tests 1 (CT1) and 2 (CT2) to assess the participant’s ability to identify the next number in a sequence while ignoring irrelevant numbers.^40^ Rey Auditory Verbal Learning Test (RAVLT) was used to assess learning and episodic verbal memory; scores are reported as RAVLTi for immediate recall of a 15-word list and RAVLTd for delayed recall after distraction. The Stroop Color and Word Test was used to assess the ability to inhibit cognitive interference,^41^ and scored using an algorithm proposed by Golden^42,43^ wherein the data of raw word and color scores were converted into Golden’s Stroop interference score (IG), using the formula with lower score means greater difficulty in inhibiting interference. We did not combine response inhibition with other executive function scores, as we observed differential directionality of correlation between KL levels and the scores of IG, and other cognitive domains (WM and VF) in HCs (Supplementary Table S1). The raw cognitive test scores were standardized into *z-scores* per cognitive domain to compare the 5 different cognitive domains between the 2 groups. These *z-scores* were calculated using the pooled mean of the overall study sample scores. For each domain, a composite *z*-score was derived by calculating the sum of the *z-scores* for the individual tests comprising that domain.

For cognitive subtyping, cluster analyses were performed in R v3.4.4 using the packages “tidyverse,” “cluster,” “factoextra,” “dendextend,” “purr,” and “NbClust.” We performed hierarchical clustering to investigate the presence of subgroups in the cognitive variates by generating a distance matrix using the Euclidean distance between the loading scores. The optimal number of clusters was determined by inspecting the corresponding dendrogram, the elbow method, and the average silhouette index.

### Analysis of Klotho Concentrations

Klotho levels were analyzed in serum samples using the commercially available human soluble α-Klotho DuoSet ELISA (cat# DY5334-05 R&D System Inc.).

### Genotyping

Genomic DNA was extracted from the blood samples of each participant using the QIAamp DNA Midi Kits (Qiagen) according to the manufacturer’s protocol. We genotyped all participants for F352V (rs9536314 T/G) which tags the KL-VS rs9536314 (F352V) using TaqMan genotyping reaction (Applied BioSystems), assay ID: C___2983037_20. DNA was amplified on a Veriti Thermal Cycler (Applied BioSystems; 95 °C for 10 minutes, followed by 40 cycles of 95 °C for 15 seconds, and 60 °C for 1 minute), and fluorescence was detected in a 7500 Fast Real‐Time PCR System (Applied BioSystems) according to the protocol of the supplier (Applied Biosystems).

### Statistical Analysis

Statistical analyses were performed using SPSS, version 28 (IBM Corp). Normality was assessed by inspecting Q-Q plots, histograms, and the Shapiro-Wilks test of normality. Between-group differences in characteristics were analyzed with analysis of variance for continuous variables, Mann-Whitney *U* tests for nonparametric data and χ2 tests for proportions. Pearson’s chi-square goodness-of-fit test was used to test for Hardy-Weinberg equilibrium (HWE) and genotype frequency distributions between study groups. Based on prior effects of Klotho on cognition concerning KL-VS heterozygosity, we also conducted a comparative analysis between individuals carrying 1 copy of the G allele of KL-VS (GT heterozygotes) vs individuals carrying zero copies of the allele (TT homozygotes).^10^ The primary outcome measures were the *z*-scores of the 5 cognitive domains, which were analyzed using linear regression, including the effect of KL-VS heterozygosity. Group comparisons of serum KL levels were performed by log-transforming Klotho levels, applying multivariable linear regression adjusting for age, gender, and KL-VS heterozygosity (TG vs TT). Associations between serum Klotho levels and cognitive scores were analyzed using univariable linear regression. The genetic association was assessed as the main effect of the disease status as well as via an interaction term between disease status (patient vs control) and KL-VS^Het+^ genotype to showcase the difference in the influence of the KL-VS genotype across the 2 subtypes of patients and the controls. Further, the influence of the KL-VS on cognitive subtypes was tested using patients and controls were estimated within the same linear regression model for each outcome, with covariates included as age, gender, and education. They were chosen based on the known potential to confound the analysis. Further, interaction terms like KL-VS^Het+^ × disease status and KL-VS^Het+^ age were added separately to analyze the influence of these covariates on cognitive scores in the combined sample analyses (SZ patients and HCs). The association between log-transformed Klotho levels and cognitive scores was examined similalry by univariable linear regression. Robust bootstrapped estimation of the standard errors with 1000 replications was used to avoid overfitting of regression models.

## Results

Demographic, genetic, and neurocognitive data for the groups examined in the study are given in Table 1. SZ patients had a similar distribution of age, gender, and education compared with controls (*P* > .05). Aligning with prior reports, most SZ patients (69.46%) represented the CD cluster.^34^ The genotype and allele frequency distributions followed the HWE in the SZ patients (χ^2^ = 0.02, *P* = .990) and controls (χ^2^ = 0.02, *P* = .990). KL-VS genotype distribution did not significantly differ between study groups (schizophrenia vs controls, c2 = 0.804, *P* = .665) or by cognitive subgroups (CD vs CD, χ^2^ = 1.03, *P* = .599) in SZ patients. The genotype frequencies of the KL-VS observed in the present Asian Indian sample were within the range of the earlier published results (allele frequency ~ 0.150; published range 0.190-0.320 for KL-VS heterozygotes, 0.010-0.032 for KL-VS homozygotes^1,10,18,45^). Kl levels were significantly lower in patients with SZ compared to HCs, median = 489.90 (IQR = 206.15-1129.90) vs 1020.00 (IQR = 230-2780) pg/mL. Similarly, the cognitive deficit subgroup of the SZ patients demonstrated lower KL levels compared to the CS subtype. As expected, all individual neurocognitive test scores were significantly lower in patients than controls. No significant differences were observed in the distribution of allele or genotype frequencies of the KL-VS variant across the patient and control group (χ^2^ = 0.804, *P* = 0.665).

**Table 1.** Distribution of Demographic Variables, Cognitive Scores, Klotho Levels and its KL-VS Gene Variant in the Study Populations

	SZ (*n* = 239)	HC (*n* = 243)	Test statistic, *P* value	CD (*n* = 166)	CS (*n* = 73)	Test statistic, *P* value
Age	35.08 (9.152)	35.31 (9.84)	0.858	35.94 (9.57)	33.29 (7.84)	0.084
Gender—*N* (% of male)	116 (48.53)	110 (45.27)	0.47, 0.523	84 (50.91)	31 (42.)	0.261
Education, years—mean (SD)	11.96 (4.05)	12.38 (4.04)	0.228	11.57 (4.10)	12.82 (3.83)	0.006*
KL-VS—MAF (G)	65 (0.136)	72 (0.148)	0.29, 0.588	47 (0.142)	14 (0.128)	0.29, 0.591
GG, *n* (%)	179 (74.90)	175 (72.02)	0.80, 0.587	122 (73.49)	57 (78.08)	1.03, 0.599
GT, *n* (%)	55 (23.01)	64 (26.34)		41 (24.70)	14 (19.18)	
TT, *n* (%)	5 (2.10)	4 (1.65)		3 (1.81)	2 (2.74)	
KL, pg/mL	489.90 [206.15-1129.90]	1020.00 [230-2780]	<0.001**	469.90 [182.40-823.65]	577.51 [231.78-1341.29]	0.001*
Attention						
CT1 (s)	91.67 (63.81)	54.49 (27.73)	8.48, <0.001	103.47 (71.25)	63.69 (18.23)	<0.001
CT2 (s)	159.16 (112.51)	111.57 (42.74)		172.59 (127.61)	127.23 (52.20)	<0.001
Processing speed						
DSST score	43.87 (17.09)	63.54 (20.61)	−9.24, <0.001**	41.61 (14.03)	49.22 (15.13)	
Executive function						
Working memory						
LNS	6.09 (2.83)	8.94 (3.43)	<0.001*	5.54 (2.71)	7.53 (3.29)	<0.001*
Verbal N-back 1 hit, accuracy (%)	49.71 (12.43)	55.90 (5.50)	<0.001*	47.71 (12.98)	54.46 (9.54)	<0.001*
Verbal N-back 2 hits, accuracy (%)	38.20 (13.20)	44.47 (9.89)	−5.12, <0.001*	36.58 (12.25)	42.05 (14.62)	<0.001*
Verbal fluency, COWA scores	8.01 (4.23)	11.00 (4.58)	<0.001*	7.30 (3.90)	8.63 (3.54)	<0.001*
Learning and episodic verbal memory						
IR	7.69 (3.09)	11.21 (2.56)	<0.001**	6.93 (2.76)	9.40 (3.13)	<0.001*
DR	7.62 (3.27)	12.81 (13.50)	−12.40, <0.001**	7.94 (6.12)	8.83 (6.25)	<0.001*
Response inhibition						
IG score	−6.78 (14.32)	3.82 (13.19)	<0.001*	−12.17 (11.05)	−5.52 (10.30)	<0.001*

SZ refers to schizophrenia; HC refers to healthy controls; DSST, digit symbol substitution test; CT, color trail test; IR refers to immediate recall; DR refers to delayed recall; IG refers to Golden’s Stroop interference score (IG); COWA refers the Controlled Oral Word Association; MAF refers to minor allele frequency.

*refers to P<0.05, and **refers to P<0.001.

Table 2 presents the linear regression coefficients that tested the association between KL-VS heterozygosity (KL-VS^Het+^) and neurocognitive measures in the study sample, considering the covariate effects. In the adjusted models, the cognitive scores were significantly and negatively associated with SZ status compared to controls for most of the domains with a moderate effect size range^44^ (β = −.30 to −.50), other than response inhibition with strong effect size, β = −.60 and delayed recall with lower magnitude, β = −.16 (Table 2).

**Table 2.** Regression Coefficients of Covariate-Adjusted Linear Regression Analyses for Cognitive Scores for Status of Schizophrenia and KL-VS^Het+^ Genotype/KL levels in an Asian Indian Sample

	Variable	Attention	Processing speed	Executive function	Response inhibition	RAVLT immediate recall	RAVLT delayed recall
	Age, years	−0.19, 0.001*	−0.23, <0.001* *	−0.07, 0.075	−0.11, 0.001*	−0.09, 0.029*	0.014, 0.795
	Gender (female vs male)	−0.08, 0.053	−0.04, 0.295	0.07, 0.072	0.01, 0.734	0.10, 0.009*	−0.07, 0.208
	Education, in years	0.10, 0.016*	0.31, <0.001**	0.03, 0.516	0.07, 0.070	0.16, <0.001*	0.12, 0.021*
	Disease status	−0.39, <0.001**	−0.44, <0.001**	−0.40, 0.001*	−0.60, 0.001*	**−**0.51, <0.001*	−0.16, 0.002*
KL-VS	GT vs GG	−0.03, 0.853	1.67, 0.453	0.09, 0.649	0.05, 0.272	−0.18, 0.658	−0.41, 0.765
	KL-VS^Het+^ × age	−0.63, 0.070	−0.10, 0.672	0.11, 0.638	0.17, 0.258	−0.69, 0.011*	0.10, 0.644
	KL-VS^Het+^ × disease status	−0.56, 0.060*	−0.07, 0.736	−0.09, 0.661	0.35, 0.046*	−0.26, 0.188	0.19, 0.269
	CD, KL-VS^Het+^ × disease status	−0.24, 0.321	−0.13, 0.608	−0.61, 0.043*	0.38, 0.043*	−0.10, 0.712	−0.09, 0.690
	CS, KL-VS^Het+^ × disease status	−0.62, 0.071	−0.11, 0.711	0.17, 0.365	0.50, 0.072	−0.27, 0.507	0.24, 0.129
Klotho	LNKL, pg/mL	0.14, 0.001*	0.13, 0.009*	0.15, 0.001*	0.10, 0.03*	0.08, 0.076	0.01, 0.723
	LNKL × age	0.20, 0.377	−0.39, 0.131	−0.23, 0.428	−0.27, 0.258	−0.55, 0.046*	−0.41, 0.059*
	LNKL × disease status	−0.35, 0.208	0.18, 0.525	−0.12, 0.617	0.72, <0.004*	0.24, 0.811	0.18, 0.389
	CD, LNKL × disease status	0.07, 0.809	0.60, 0.131	−0.90, 0.028*	0.29, 0.313	0.09, 0.824	0.21, 0.379
	CS, LNKL × disease status	−0.10, 0.945	0.09, 0.961	0.32, 0.748	0.31, 0.157	0.38, 0.278	0.28, 0.436

DS refers to disease status patient vs control; CD refers to cognitive deficit; CS refers to cognitively spared; model was adjusted for age; LNKL refers to natural log-transformed serum Klotho levels in pg/mL; gender, and education; disease refers to status of schizophrenia (yes/no); KL-VS^Het+^ refers to the genotype comparison between s. FF s; under response inhibition, the analyses have been done using Golden’s Stroop interference score (IG); results are presented as β, or standardized beta coefficient, and *P* values.

*,**refers to statistics with P value <0.05, and <0.001, respectively.

### Attention

No main effect was observed for KL-VS^Het+^ on attention scores when analyzed in HCs (Supplementary Table S1) and the combined samples (Table 2). When tested for interaction effects, there was a borderline significant trend of negative interaction observed between schizophrenia diagnosis and KL-VS^Het+^ on attention *z*-scores; β = −.56, *P* = .060 (Table 2). Similarly, age negatively modulated the genetic association of KL-VS^Het+^ with attention (β = −.62, *P* = .062). There was a weak positive correlation between Kl levels and attention scores (β = −.14, *P* = .002), but without influencing disease status/age as observed for KL-VS^Het+^ status.

### Processing Speed

No main effects of KL-VS^Het+^ were observed for the processing speed scores (DSST) (Table 2). However, a weak but positive interaction was observed with KL levels and DSST (β = .13, *P* = .009).

### Executive Function

For executive function, the combined scores of WM and VF were tested for the genetic influence of KL-VS (Table 2). The CD cluster exhibited a strong negative interaction between SZ status and the KL-VS heterozygosity β = −.61, *P* = .043, and the effect was further enhanced when analyzed at the protein levels of KL, β = −.91, *P* = .028.

Similarly, when analyzed individually, the WM scores exhibited strong negative directionality for Kl levels by disease status (β = −.90, *P* = .008), which was specific to the CD cluster (Supplementary Table S2). However, the effects remained limited to the protein levels (β = −.90 and .79, *P* < .001, respectively). On the contrary, for VF, the reversed effect of KL across disease status remained limited to the genetic levels (KL-VS^Het+^) in the CD cluster, β = −.91, *P* = .02.

### Response Inhibition

Regarding IG scores of the Stroop effect of response inhibition, opposing trends of KL and KL-VS were observed compared to the other cognitive variables in the study. There was a nonsignificant trend of negative association between IG scores and KL-VS^Het+^ in controls (Supplementary Table S1). Moreover, there was also an unanticipated positive interaction between disease status (presence of schizophrenia) and KL-VS^Het+^, β = .35, *P* = .046, limited to the CD cluster, β = .35, *P* = .046. A similar but more potent effect of disease status was also evident at KL protein levels, β = .72, <.004, that could not be replicated for the CD cluster effect.

### Learning and Episodic Memory

There was no significant main effect of KL-VS^Het+^ on the scores of RAVLTi and RAVLTd. However, a strong negative interaction effect could be established between KL and age for RAVLTi at both genetic (KL-VS^Het+^ × age, β = −.61, *P* = .011), and protein levels (KL levels × age, β = .55, *P* = .046).

## Discussion

The present study reports a unique investigation of the effects of a well-reported cognition-enhancing gene, Klotho, at both its genetic and protein levels on cognitive impairment in schizophrenia. It is also important to mention that, though there are very few relevant Indian studies reported on alteration in the expression of Klotho in schizophrenia in India^27^ with a few key reviews presented by the same group of authors on the role of Klotho in schizophrenia.^26–28^ However, to the best of our knowledge, this is the first Asian Indian study to evaluate the influence of the genetic contribution of Klotho on cognitive function in schizophrenia. This consideration becomes vital given that there is almost nil representation of South Asian ancestry in the makeup of previously reported samples.^23,33^ Notably, the observed minor allele frequency of the rs9536314 loci, the key locus underlying the KL-VS variants, was 14.8%, similar to that at the global population level and European descent (15.05%), but was slightly lower than that reported for South Asians.^45^ Notably, we could confirm the adverse effect of KL-VS heterozygosity on cognition (executive function) in the CD subtype of SZ patients, as also reported by Morar et al.^10^ in the WAFSS cohort. Interestingly, the same effect could be replicated at KL protein levels with stronger effect size (β = −.90). Hence, as deemed before,^10,22^ inclusion of circulatory levels of Klotho aided in enhancing the reliability of the unexpected effect of KL on cognition in SZ patients. The distinct interaction effects observed in the CD subtype of cognition also reaffirm the utility of cognitive subtyping for delineating genetic etiology.^46^ However, our findings do differ from the earlier report on SZ^10^ concerning the domains of cognition where the effects of KL-VS could be demonstrated (combined scores of WM and VF vs episodic verbal memory,^10^ respectively). These disparities of the gene variant on differential neuronal underpinnings of cognitive decline could be partially attributed to the ancestry-specific genetic effects as also observed for APOE alleles,^47^ and already known racial disparities in KL-VS allele frequency distribution.^23,33^ Further, we also failed to replicate the significant main effect of KL-VS heterozygosity on any of the studied cognitive variables, which has also been an important recent observation.^23^ Overall, the lack of significant main effect of KL-VS^Het+^ on cognition, with an unanticipated lack of influence of the variant on KL^16,22,24,46^ protein levels (controls), and overall weaker positive correlations between KL levels and cognition^30^ (attention, WM, VF, processing speed) strengthen the notion that KL-VS^Het+^ alone might not be a significant modulator of cognition alone and may have strong confounding effects of age,^10,25^ environmental risk factors,^48,49^ and epigenetics.^50^ Moreover, our results also demonstrate a strong negative interaction between age and KL-VS^Het+^ for domains of immediate and delayed recall, and the same was also mediated at protein levels for the former.

One of the unique observations arising from the study was the cognitive dissociation evident concerning the influence of Klotho levels and heterozygosity of its KL-VS variant on different indices of executive function. The KL-VS^Het+^ was parallelly protective for response inhibition and risk conferring for attention, VF, and WM in SZ patients. We find comparability in our findings with that of the previously reported cognitive dissociations observed in the effects of APOE ε4 carriers for different categories of memory.^51^ Taken together, dissociable effects of the Klotho KL-VS variant on different cognitive domains suggest differential neural underpinnings of the effect of the genotype necessitating the need to understand the putative partners/receptors of Klotho in such domain-specific cognitive processes as proposed by Morar et al.^10^

Aligning with the findings of Morar et al.,^10^ our observation on the reversed directionality of the KL-VS heterozygotes performing worse than noncarriers on tasks of WM and VF, especially in the cognitively deficient cluster, is further extended by the similar associations observed at KL protein levels. We agree with the notion put forth by Morar et al.^10^ about the modifying influence of the pathological milieu of SZ, underlying the reversed directionality of KL-VS^Het+^. We substantiate the same notion by considering SZ as a heterogeneous disease driven by multiple causative factors with complex interplays between genetic and environmental risk factors/exposures such as trace elements,^52,53^ and plausible interaction effects between genetic variants such as the one reported between Klotho KL-VS and APOE influencing the cognitive outcomes in Alzheimer’s.^54^ Hence, another angle to be explored remains the influence of interaction between Kl-VS and other etiological alleles on cognition. The unexpected reversed directionality of the association between Klotho and cognition also needs consideration given the recently reported observation on aged nonhuman primates,^24^ wherein authors demonstrated beneficial outcomes of administration of Klotho on memory to be restricted to its lower rather than higher doses,^24^ and with a nonlinear association established between Klotho levels and cognitive outcomes like DSST.^55^

Regarding the genotype distribution across patients with SZ and controls, we could not implicate the KL-VS variant with the disease status of schizophrenia, also coinciding with the findings of Morar et al.^10^ and the lack of evidence from the multistage SZ genome-wide association study (GWAS) not supporting any genome-wide significant signals from the Klotho harboring chromosome.^56,57^ As mentioned earlier, there was also a lack of influence of KL-VS^Het+^ on KL protein levels in controls (data not shown), with slight evidence of heterozygosity-associated KL elevation in patients. Hence, contrary to the genetic distributions of KL-VS, there was considerable downregulation of KL levels observed between SZ patients and controls. Given the factor that our data were derived from chronic SZ patients, we add to the dynamic relationship reported between schizophrenia and Klotho, with already reported significantly lower as well as elevated levels in patients with schizophrenia being admitted to hospitals reporting acute exacerbation for their psychotic symptoms.^29,30^

Regarding the genotype distribution across patients with SZ and controls, we could not implicate the KL-VS variant with the disease status of schizophrenia, also coinciding with the findings of Morar et al.^10^ and the lack of evidence from the multistage SZ GWAS not supporting any genome-wide significant signals from the Klotho harboring chromosome. On the contrary to genetic distributions of KL-VS, there was considerable downregulation of KL levels observed between SZ patients and controls. Our data from chronic SZ patients add to the dynamic relationship reported between schizophrenia and Klotho, with significantly lower as well as elevated levels observed in patients with schizophrenia being admitted to hospitals reporting acute exacerbation for their psychotic symptoms.^29,30^ As mentioned earlier, KL-VSHet^+^ also did not influence KL protein levels in controls, with slight evidence of heterozygosity-associated KL elevation in patients.

Overall, the missed association with SZ risk but with certain domains of cognition, further restricted to the cognitively deficient subtype, reaffirms the utility of cognitive phenotyping subtle genetic effects underlying complex and heterogenous traits like SZ. The major strength of the present study is the wider breadth of cognitive tests, compared to previous studies. The study also addressed the effect of circulating Klotho in blood along with the KL-VS genetic variant to aid in a better understanding of Klotho’s effect on cognition in the context of SZ.

Overall, these dissociable patterns of association indicate the need to exert caution over accepting any generalized effect of Klotho gene variants on cognition and considering the plausible population- and domain-specific discordant effects. This notion becomes relevant given the generalized therapeutic benefits deemed to be associated with the elevation of wild-type Klotho levels or its activities deemed to confer an advantage against neurological and other cognitive disorders including Alzheimer’s disease.

## Supplementary Material

sgae030_suppl_Supplementary_Materials

## References

[1] Mihaljević-Peleš A , Bajs Janović M, Šagud M, Živković M, Janović S, Jevtović S. Cognitive deficit in schizophrenia: an overview. Psychiatr Danub. 2019;31:139–142. 31158113

[2] Nuechterlein KH , Green MF, Kern RS, et al The MATRICS Consensus Cognitive Battery, part 1: test selection, reliability, and validity. Am J Psychiatry. 2008;165:203–213. https://doi.org/10.1176/appi.ajp.2007.07010042 18172019

[3] Harvey PD , Bosia M, Cavallaro R, et al Cognitive dysfunction in schizophrenia: an expert group paper on the current state of the art. Schizophr Res Cogn. 2022;29:100249. https://doi.org/10.1016/j.scog.2022.100249 35345598 PMC8956816

[4] Kroken RA , Løberg EM, Drønen T, et al A critical review of pro-cognitive drug targets in psychosis: convergence on myelination and inflammation. Front Psychiatry. 2014;5:11. https://doi.org/10.3389/fpsyt.2014.00011 24550848 PMC3912739

[5] Minzenberg MJ , Yoon JH, Soosman SK, Carter CS. Altered brainstem responses to modafinil in schizophrenia: implications for adjunctive treatment of cognition. Transl Psychiatry. 2018;8:58. https://doi.org/10.1038/s41398-018-0104-z 29507283 PMC5838154

[6] Braff DL , Freedman R, Schork NJ, Gottesman II. Deconstructing schizophrenia: an overview of the use of endophenotypes in order to understand a complex disorder. Schizophr Bull. 2007;33:21–32. https://doi.org/10.1093/schbul/sbl049 17088422 PMC2632293

[7] Donati FL , D’Agostino A, Ferrarelli F. Neurocognitive and neurophysiological endophenotypes in schizophrenia: an overview. Biomark Neuropsychiatry. 2020;3:100017. https://doi.org/10.1016/j.bionps.2020.100017 37293386 PMC10249673

[8] McCutcheon RA , Keefe RSE, McGuire PK. Cognitive impairment in schizophrenia: aetiology, pathophysiology, and treatment. Mol Psychiatry. 2023;28:1902–1918. https://doi.org/10.1038/s41380-023-01949-9 36690793 PMC10575791

[9] Egan MF , Goldberg TE, Kolachana BS, et al Effect of COMT Val108/158 Met genotype on frontal lobe function and risk for schizophrenia. Proc Natl Acad Sci U S A. 2001;98:6917–6922. https://doi.org/10.1073/pnas.111134598 11381111 PMC34453

[10] Morar B , Badcock JC, Phillips M, Almeida OP, Jablensky A. The longevity gene Klotho is differentially associated with cognition in subtypes of schizophrenia. Schizophr Res. 2018;193:348–353. https://doi.org/10.1016/j.schres.2017.06.054 28673754

[11] Lally J , MacCabe JH. Personalised approaches to pharmacotherapy for schizophrenia. BJPsych Adv. 2016;22:78–86. https://doi.org/10.1192/apt.bp.114.013433

[12] Stephan KE , Baldeweg T, Friston KJ. Synaptic plasticity and dysconnection in schizophrenia. Biol Psychiatry. 2006;59:929–939. https://doi.org/10.1016/j.biopsych.2005.10.005 16427028

[13] Forsyth JK , Lewis DA. Mapping the consequences of impaired synaptic plasticity in schizophrenia through development: an integrative model for diverse clinical features. Trends Cogn Sci. 2017;21:760–778. https://doi.org/10.1016/j.tics.2017.06.006 28754595 PMC5610626

[14] Conn KA , Burne THJ, Kesby JP. Subcortical dopamine and cognition in schizophrenia: looking beyond psychosis in preclinical models. Front Neurosci. 2020;14:542. https://doi.org/10.3389/fnins.2020.00542 32655348 PMC7325949

[15] Cheng W , van der Meer D, Parker N, et al Shared genetic architecture between schizophrenia and subcortical brain volumes implicates early neurodevelopmental processes and brain development in childhood. Mol Psychiatry. 2022;27:5167–5176. https://doi.org/10.1038/s41380-022-01751-z 36100668

[16] Dubal DB , Yokoyama JS, Zhu L, et al Life extension factor Klotho enhances cognition. Cell Rep. 2014;7:1065–1076. https://doi.org/10.1016/j.celrep.2014.03.076 24813892 PMC4176932

[17] Ananya FN , Ahammed MR, Lahori S, et al Neuroprotective role of Klotho on dementia. Cureus. 2023;15:e40043. https://doi.org/10.7759/cureus.40043 37425590 PMC10324629

[18] Arking DE , Krebsova A, Macek M, et al Association of human aging with a functional variant of Klotho. Proc Natl Acad Sci U S A. 2002;99:856–861. https://doi.org/10.1073/pnas.022484299 11792841 PMC117395

[19] de Vries CF , Staff RT, Harris SE, et al Klotho, APOEε4, cognitive ability, brain size, atrophy, and survival: a study in the Aberdeen Birth Cohort of 1936. Neurobiol Aging. 2017;55:91–98. https://doi.org/10.1016/j.neurobiolaging.2017.02.019 28431289

[20] Guo JY , Huhtaniska S, Miettunen J, et al Longitudinal regional brain volume loss in schizophrenia: relationship to antipsychotic medication and change in social function. Schizophr Res. 2015;168:297–304. https://doi.org/10.1016/j.schres.2015.06.016 26189075 PMC4604250

[21] Mengel-From J , Soerensen M, Nygaard M, McGue M, Christensen K, Christiansen L. Genetic variants in Klotho associate with cognitive function in the oldest old group. J Gerontol A Biol Sci Med Sci. 2016;71:1151–1159. https://doi.org/10.1093/gerona/glv163 26405063 PMC4978356

[22] Grøntvedt GR , Sando SB, Lauridsen C, et al Association of Klotho protein levels and KL-VS heterozygosity with Alzheimer disease and amyloid and tau burden. JAMA Netw Open. 2022;5:e2243232. https://doi.org/10.1001/jamanetworkopen.2022.43232 36413367 PMC9682425

[23] de Vries CF , Staff RT, Noble KG, et al; Pediatric Imaging, Neurocognition and Genetics Study. Klotho gene polymorphism, brain structure and cognition in early-life development. Brain Imaging Behav. 2020;14:213–225. https://doi.org/10.1007/s11682-018-9990-1 30393836 PMC6588504

[24] Castner SA , Gupta S, Wang D, et al Longevity factor Klotho enhances cognition in aged nonhuman primates. Nat Aging. 2023;3:931–937. https://doi.org/10.1038/s43587-023-00441-x 37400721 PMC10432271

[25] Müller BW , Hinney A, Scherbaum N, et al Klotho KL-VS haplotype does not improve cognition in a population-based sample of adults age 55-87 years. Sci Rep. 2021;11:13852. https://doi.org/10.1038/s41598-021-93211-x 34226614 PMC8257625

[26] Birdi A , Tomo S, Sharma M, et al Association of Klotho with neuropsychiatric disorder: a meta-analysis. Indian J Clin Biochem. 2024;39:429–437. https://doi.org/10.1007/s12291-023-01132-5 39005867 PMC11239616

[27] Birdi A , Kumar PK, Nebhinani N, et al Association of circulatory Klotho levels and its expression with miRNA-339 in patients with schizophrenia. Behav Brain Res. 2023;445:114359. https://doi.org/10.1016/j.bbr.2023.114359 36842554

[28] Birdi A , Tomo S, Yadav D, et al Role of Klotho protein in neuropsychiatric disorders: a narrative review. Indian J Clin Biochem. 2023;38:13–21. https://doi.org/10.1007/s12291-022-01078-0 36684492 PMC9852376

[29] Turkmen BA , Yazici E, Erdogan DG, Suda MA, Yazici AB. BDNF, GDNF, NGF and Klotho levels and neurocognitive functions in acute term of schizophrenia. BMC Psychiatry. 2021;21:562. https://doi.org/10.1186/s12888-021-03578-4 34763683 PMC8588660

[30] Xiong JW , Zhan JQ, Luo T, et al Increased plasma level of longevity protein Klotho as a potential indicator of cognitive function preservation in patients with schizophrenia. Front Neurosci. 2020;14:610. https://doi.org/10.3389/fnins.2020.00610 32612508 PMC7308714

[31] Kawano K , Ogata N, Chiano M, et al Klotho gene polymorphisms associated with bone density of aged postmenopausal women. J Bone Miner Res Off J Am Soc Bone Miner Res. 2002;17:1744–1751. https://doi.org/10.1359/jbmr.2002.17.10.1744 12369777

[32] Majumdar V , Christopher R. Association of exonic variants of Klotho with metabolic syndrome in Asian Indians. Clin Chim Acta. 2011;412:1116–1121. https://doi.org/10.1016/j.cca.2011.02.034 21376714

[33] Xu X , Liang X, Hu G, Zhang J, Lei H. Renal function and Klotho gene polymorphisms among Uygur and Kazak populations in Xinjiang, China. Med. Sci. Mon. 2015;21:44–51. https://doi.org/10.12659/MSM.891213 PMC4292765 25556925

[34] Kübler U. Structured clinical interview for DSM-IV (SCID). In: Gellman MD, Turner JR, eds. Encyclopedia of Behavioral Medicine. Springer New York; 2013:1919–1920. https://doi.org/10.1007/978-1-4419-1005-9_66

[35] Cannon TD , Huttunen MO, Lonnqvist J, et al The inheritance of neuropsychological dysfunction in twins discordant for schizophrenia. Am J Hum Genet. 2000;67:369–382. https://doi.org/10.1086/303006 10880296 PMC1287184

[36] Hallmayer JF , Kalaydjieva L, Badcock J, et al Genetic evidence for a distinct subtype of schizophrenia characterized by pervasive cognitive deficit. Am J Hum Genet. 2005;77:468–476. https://doi.org/10.1086/432816 16080121 PMC1226211

[37] Jaeger J. Digit symbol substitution test: the case for sensitivity over specificity in neuropsychological testing. J Clin Psychopharmacol. 2018;38:513–519. https://doi.org/10.1097/JCP.0000000000000941 30124583 PMC6291255

[38] David W. WMS-R: Wechsler Memory Scale-Revised. Psychological Corporation; 1997.

[39] Owen AM , McMillan KM, Laird AR, Bullmore E. N-back working memory paradigm: a meta-analysis of normative functional neuroimaging studies. Hum Brain Mapp. 2005;25:46–59. https://doi.org/10.1002/hbm.20131 15846822 PMC6871745

[40] D’Elia LF , Satz P, Uchiyama CL, White T. Color Trails Test (CTT). Odessa, FL: Psychological Assessment Resources; 1996.

[41] Stroop JR. Studies of interference in serial verbal reactions. J Exp Psychol. 1935;18:643–662. https://doi.org/10.1037/h0054651

[42] Golden CJ. Stroop Color and Word Test: A Manual for Clinical and Experimental Uses. Stoelting Co.; 1978.

[43] Golden CJ. A Manual for the Clinical and Experimental Use of the Stroop Color and Word Test. Stoelting Company; 1978.

[44] Cohen J. Statistical Power Analysis for the Behavioral Sciences. 2nd ed. L. Erlbaum Associates; 1988.

[45] rs9536314. National Center for Biotechnical Information. https://www.ncbi.nlm.nih.gov/snp/?term=rs9536314.

[46] Yokoyama JS , Marx G, Brown JA, et al Systemic Klotho is associated with Klotho variation and predicts intrinsic cortical connectivity in healthy human aging. Brain Imaging Behav. 2017;11:391–400. https://doi.org/10.1007/s11682-016-9598-2 27714549 PMC5382127

[47] Granot Hershkovitz E , Tarraf W, Kurniansyah N, et al *APOE* alleles’ association with cognitive function differs across Hispanic/Latino groups and genetic ancestry in the study of Latinos‐investigation of neurocognitive aging (HCHS/SOL). Alzheimers Dement. 2021;17:466–474. https://doi.org/10.1002/alz.12205 33155766 PMC8016734

[48] Matsubara T , Miyaki A, Akazawa N, et al Aerobic exercise training increases plasma Klotho levels and reduces arterial stiffness in postmenopausal women. Am J Physiol Heart Circ Physiol. 2014;306:H348–H355. https://doi.org/10.1152/ajpheart.00429.2013 24322608

[49] Prather AA , Epel ES, Arenander J, et al Longevity factor Klotho and chronic psychological stress. Transl Psychiatry. 2015;5:e585–e585. https://doi.org/10.1038/tp.2015.81 26080320 PMC4490291

[50] Rubinek T , Shulman M, Israeli S, et al Epigenetic silencing of the tumor suppressor Klotho in human breast cancer. Breast Cancer Res Treat. 2012;133:649–657. https://doi.org/10.1007/s10549-011-1824-4 22042362

[51] Zokaei N , Čepukaitytė G, Board AG, Mackay CE, Husain M, Nobre AC. Dissociable effects of the apolipoprotein-E (APOE) gene on short- and long-term memories. Neurobiol Aging. 2019;73:115–122. https://doi.org/10.1016/j.neurobiolaging.2018.09.017 30342272 PMC6261846

[52] Modabbernia A , Arora M, Reichenberg A. Environmental exposure to metals, neurodevelopment, and psychosis. Curr Opin Pediatr. 2016;28:243–249. https://doi.org/10.1097/MOP.0000000000000332 26867166

[53] Ray A , Birdi A, Nebhinani N, et al Correlation between severity of schizophrenia with certain trace elements and TNF-α gene expression and its circulatory level in the population of Western India. Biol Trace Elem Res. 2024. https://doi.org/10.1007/s12011-024-04301-6 38995436

[54] Kirkpatrick B , Messias E, Harvey PD, Fernandez-Egea E, Bowie CR. Is schizophrenia a syndrome of accelerated aging? Schizophr Bull. 2008;34:1024–1032. https://doi.org/10.1093/schbul/sbm140 18156637 PMC2632500

[55] Wu Y , Lei S, Li D, Li Z, Zhang Y, Guo Y. Relationship of Klotho with cognition and dementia: results from the NHANES 2011-2014 and Mendelian randomization study. Transl Psychiatry. 2023;13:337. https://doi.org/10.1038/s41398-023-02632-x 37914711 PMC10620156

[56] Schizophrenia Working Group of the Psychiatric Genomics Consortium. Biological insights from 108 schizophrenia-associated genetic loci. Nature. 2014;511:421–427. https://doi.org/10.1038/nature13595 25056061 PMC4112379

[57] Kuro-o M , Matsumura Y, Aizawa H, et al Mutation of the mouse Klotho gene leads to a syndrome resembling ageing. Nature. 1997;390:45–51. https://doi.org/10.1038/36285 9363890

