# Supplementary material for: Associations Between Klotho Levels, KL-VS Heterozygosity and Cognition in Schizophrenia
Source: Schizophr Bull Open. 2024 Dec 25;6(1):sgae030. doi: 10.1093/schizbullopen/sgae030 (PMC11904889; doi:10.1093/schizbullopen/sgae030)
Supplement: sgae030_suppl_Supplementary_Materials [file sgae030_suppl_supplementary_materials.docx]

**Supplementary Table 1. Main effects of KL-VSHet+ and KL levels on cognitive scores in controls**

| **Variable** | **Attention** | **Processing Speed** | **Working memory** | **Verbal Fluency** | **Learning and memory** | | **Response inhibition** |
| --- | --- | --- | --- | --- | --- | --- | --- |
|  |  |  |  |  | **RAVLTir** | **RAVLTdr** |  |
| KL-VS Het^+^ | 0.22, 0.140 | 1.12, 0.678 | 0.03, 0.683 | 0.33, 0.630 | 0.17, 0.671 | -1.52, 0.263 | -1.48, 0.412 |
| Ln Klotho | -0.02, 0.742 | 0.01, 0.935 | **0.15, 0.023*** | 0.08, 0.226 | -0.08, 0.363 | **0.12, 0.035*** | **-0.18, 0.002*** |

**Supplementary Table 2.** Regression coefficients of association analyses between KL-VE Het+ , KL levels and scores of working memory and verbal fluency

|  | **Variable** | **Executive function** | **Working memory** | **Verbal Fluency** |  |  |  |
| --- | --- | --- | --- | --- | --- | --- | --- |
|  | Age, years | -0.07, 0.075 | **-0.09, 0.043*** | -0.02, 0.44 |  |  |  |
|  | Gender (female vs. male) | 0.07, 0.072 | **0.10, 0.015*** | -0.33, 0.43 |  |  |  |
|  | Education, in years | 0.03, 0.516 | 0.01, 0.782 | -0.08, 0.21 |  |  |  |
|  | Disease status | **-0.40, 0.001*** | **-0.30, 0.001*** | **-0.40, 0.02*** |  |  |  |
| *KL-VS*  *Status Het^+^* | *GT vs. GG* | 0.09, 0.649 | 0.04, 0.859 | 0.25, 0.609 |  |  |  |
|  | KL-VS^Het+^ x age | 0.11, 0.638 | 0.01, 0.975 | -0.08, 0.653 |  |  |  |
|  | KL-VS^Het+^ x disease status | -0.09, 0.661 | -0.09, 0.780 | -0.08, 0.703 |  |  |  |
|  | CD, KL-VS^Het+^ x disease status | -**0.61, 0.043*** | -0.21, 0.529 | **-0.91, 0.02*** |  |  |  |
|  | CS, KL-VS^Het+^ x disease status | 0.17, 0.365 | 0.18, 0.581 | 0.16, 0.407 |  |  |  |
| *Klotho levels-VS* | *LNKL, pg/ml* | **0.15, 0.001*** | 0.07, 0.154 | **0.20, <0.01*** |  |  |  |
|  | LNKL x age | -0.23, 0.428 | -0.04, 0.903 | -0.26, 0.379 |  |  |  |
|  | LNKL x disease status | -0.12, 0.617 | **-0.90, 0.008*** | 0.11, 0.592 |  |  |  |
|  | CD, KL x disease status | **-0.90, 0.028*** | **-0.79, 0.056*** | -0.46, 0.166 |  |  |  |
|  | CS, KL x disease status | 0.32, 0.748 | -0.19, 0.577 | 0.46, 0.113 |  |  |  |
